# Use of Electrocautery for Coagulation and Wound Complications in Caesarean Sections

**DOI:** 10.1155/2014/602375

**Published:** 2014-07-20

**Authors:** Cristiane M. Moreira, Eliana Amaral

**Affiliations:** ^1^Department of Obstetrics and Gynaecology (PUCCAMP), Avenida Jonh Boind Dunlop s/n, 13100 Campinas, SP, Brazil; ^2^Department of Obstetrics and Gynaecology (UNICAMP), Rua Alexander Fleming, 101 Cidade Universitária, 13083-881 Campinas, SP, Brazil

## Abstract

*Objective*. To evaluate the safety of electrocautery for coagulation during Caesarean sections. *Study Design*. A randomized, controlled, clinical pilot study was performed at a university maternity hospital. After admission for delivery and decision to perform a C-section, volunteers were randomized to either the intervention group (use of electrocautery for coagulation) or nonintervention group. The women were examined at the time of postpartum discharge (day 3), at days 7 to 10, and again at days 30 to 40 for signs of infection, hematoma, seroma, or dehiscence. Data were analyzed using an intention-to-treat analysis, and risk ratios were calculated. *Results*. No significant differences were found between the two groups. Only 2.8% of patients in the intervention group developed surgical wound complications during hospitalization. However, 7 to 10 days following discharge, these rates reached 23.0% and 15.4% in the intervention and nonintervention groups, respectively (RR = 1.50, 95% CI = 0.84–2.60). *Conclusion*. Further studies should confirm whether the use of electrocautery for coagulation does not increase the risk of surgical wound complications in patients undergoing Caesarean sections.

## 1. Introduction

The surgical technique for Caesarean sections (C-sections) has undergone multiple changes aimed at improving patient outcomes. Among these changes, the use of electrocautery for obstetrical procedures was introduced according to recommendations arising from developments in general surgery [1].

Electrosurgery is expected to reduce the surgical duration because of beneficial homeostatic interventions. However, electrosurgery is also associated with potential complications including internal and external burns, seromas, and surgical scar infections [1–4]. Previous studies have suggested that infectious complications are a cause for concern [5, 6]. However, in a randomized study of oncologic patients who underwent laparotomy in which electrocautery was used for tissue cutting and hemostasis, no differences were found between the electrocautery and control group [7].

Nevertheless, no studies have been conducted on the possible complications associated with the use of surgical electrocautery in pregnant women. The United Kingdom's National Health System manual, which is based on available evidence, reports that there are no data on which to recommend the use of electrocautery in C-sections [8]. This institution considers that current evidence is insufficient to recommend the use of electrocautery in C-sections [9].

On the other hand, there are no reports of adverse effects associated with the use of electrocautery in newborns, even in intrauterine fetal surgery in high-voltage electric currents are used for periods of up to 30 minutes [10, 11].

In view of the reported increase in the performance of C-sections and use of electrocautery combined with the scarcity of pertinent data, a pilot study was performed to evaluate the efficacy and safety of the use of electrocautery for coagulation on surgical wound healing in patients undergoing C-section.

## 2. Material and Methods

We performed a randomized, controlled, clinical pilot study on the use of electrocautery for coagulation in C-sections. Because no previous studies have been performed on this topic, sample size calculation was based on a presumed 15.0% increase in surgical wound complications with the use of electrocautery for coagulation, a 33.0% prevalence of C-section deliveries in the maternity hospital in which the study was performed, and a 14.4% postdischarge infection rate [12]. An additional 20.0% was added to compensate for patients who may be lost to follow-up during the study, totaling a sample of 224 women.

Women admitted for delivery at the obstetrical unit of a university maternity teaching hospital in Campinas, State of São Paulo, Brazil, who had received prenatal care and who had no hemostatic problems, were invited to participate in the study. Admission to the study was dependent on the need to perform a C-section, which was determined by the obstetrical team at any point during labour. Patients in whom a C-section was indicated as an emergency procedure (e.g., for treatment of placental abruption), patients who have used antibiotics in the 30 days preceding delivery, and patients who had undergone more than one previous C-section were excluded from the study. The women who were invited to participate in the study received information regarding the procedures to be carried out and signed an informed consent form before the mode of delivery was defined.

When a C-section was indicated, admission criteria were checked and informed consent was obtained. The next step involved randomization to one of two groups: patients who did and did not undergo the use of electrocautery. This was accomplished by opening the next in a series of 224 brown envelopes available at the delivery room. Those envelopes indicated to which of the two groups each woman was assigned according to the randomization list generated by the SAS statistical software program version 9.1. The operating room nurse sequentially opened the envelopes.

If the use of electrocautery was required in patients allocated to the nonintervention group, this protocol deviation was to be annotated; however, no such situation occurred during the study period. All patients received prophylactic antibiotic therapy during delivery, as a single dose of cephalothin after the umbilical cord had been clamped.

After the procedure had been performed, the patient was blindly reevaluated by the investigator on the day of hospital discharged (postpartum day 3), at days 7 to 10, and at days 30 to 40. At each evaluation, the surgical scar was examined for signs of infection, hematoma, seroma, or dehiscence. Neither the volunteers nor the investigator who performed the evaluation at the three above-mentioned time points were aware of treatment. The patient was discontinued from the study if she was found to have an infection at any site other than the C-section scar.

The results were analyzed using an intention-to-treat approach. The chi-square test and Fisher's exact test were used for categorical variables, and the Mann-Whitney test was used for interval variables. Risk ratio and correspondent 95% confidence intervals were calculated. The database was constructed using Epi Info 6, version 1.0. The statistical analysis was performed using the SAS software package, version 9.1.

The surgical technique and the appropriate use of electrocautery were part of a preparatory training program for surgeons (residents, contracted physicians, and faculty). This program included a discussion session and distribution of written material. Clinical evaluations during follow-up period were carried out by the same investigator together with another physician in all cases, and in any cases of discord, a third observer was consulted. All collected data were entered into the database and checked in duplicate.

The study protocol was approved by the Internal Review Board of the School of Medical Sciences, University of Campinas (UNICAMP) under approval number 1036/2008 as well as by the Internal Review Board of the Celso PierroHospital and Maternity Home. All participants signed an informed consent form [13].

## 3. Results

**Figure 1:**
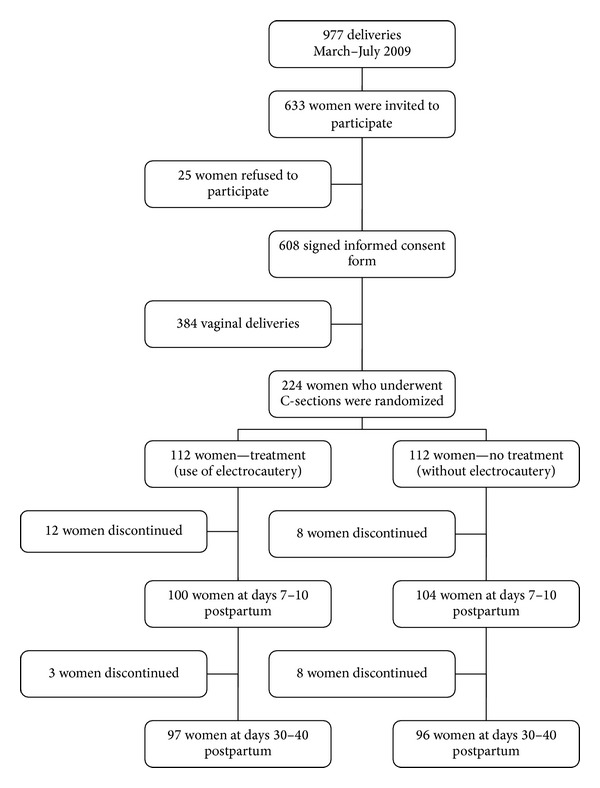
Flow chart of admission to and exclusion from the study.

Between March and July 2009, a total of 977 deliveries were performed and 633 women were invited to participate in the study prior to establishing the type of delivery. Of these women, 25 refused to participate (15 due to difficulty in attending scheduled follow-up visits and 10 for various other reasons). Of the 608 women who signed as informed consent form, the 224 underwent C-section were randomized to the two above-described groups. Four were eliminated from analysis (two due to use of prophylactic antibiotics after prolonged urethral catheterization and two due to antibiotic use for postpartum urinary infection). No electrocautery protocol deviation occurred. Fifteen women in the intervention group and 16 in the nonintervention group did not return for the 30- to 40-day evaluation; therefore, 97 patients in the intervention group and 96 in the nonintervention group completed the protocol, including the late evaluation visit at days 30 to 40 (Figure 1).

There were no statistical differences in demographic characteristics, parity, prenatal care, or weight between the two groups. The mean age of the women was 24 years, and they had a mean of 10 years of schooling. In terms of marital status, 70% lived with a partner; of those who lived alone, 96% had a steady sexual partner. The patients had attended as average of nine prenatal consultations, had an initial mean body mass index of 25.3 kg/m^2^, and had gained a mean of 12 kg during pregnancy. Most patients (60%) were in their second pregnancy. One-third of the women in both groups (34%) had already undergone one C-section (Table 1).

In 80% of cases, performance of the C-section was indicated during labor by the physician for reasons primarily associated with fetal compromise (26%) and failed labor induction (11%). There were 14 cases and 17 cases of preeclampsia in the intervention nonintervention groups, respectively. The incidence of diabetes in both groups was 1%.

All patients underwent spinal anaesthesia with the exception of one woman in whom general anaesthesia was necessary. In both groups, 71% of the procedures were performed by a year 2 resident with the assistance of year 1 resident under staff supervision. Intraoperative complications included maternal supraventricular tachycardia, change from cephalic to breech presentation, bladder lesions, epidural block failure, and termination of the anaesthetic effect prior to the end of surgery; these complications predominated in the nonintervention group.

The total time of the surgical procedure (from anaesthesia to completion of the C-section) was similar in both groups: 100 and 105 minutes in the intervention and nonintervention groups, respectively (*P* = 0.54). The surgeon's training was not associated with the incidence of surgical wound complications (*P* = 0.57). Neonatal findings were similar in both groups, and no newborn infants had cardiac arrhythmia at birth (data not shown).

During hospitalization, only 2.8% of the women developed a surgical scar complication (hematoma, seroma, dehiscence, or signs of infection), and all of these complications occurred in the electrocautery group. Three women developed signs of infection, which were associated with a seroma in one patient (Table 2). In the evaluation performed between days 7 and 10, when stitches were removed, a significant increase in the complications rate was found: 23.0% and 15.4% in the intervention and nonintervention groups, respectively. Seroma was the most common complication (17.0% and 11.5%, resp.), followed by signs of infection, dehiscence, and hematoma. There was no difference in the distribution of complications between the two groups (Table 3).

Few women reported complications at the 30- to 40-day evaluation (Table 4). At the time of stitch removal (days 7–10), a risk ratio of 1.5 was found for complications compared with discharge, which was not statistically significant (95% CI = 0.84–2.60). At the final postpartum check-up, 23.0% and 18.8% of the women in the intervention and nonintervention groups, respectively, were found to have some form of surgical wound complication (RR = 1.21; 95% CI = 0.69–2.11). Nevertheless, there was no increase in the accumulated risk of developing a complication (seroma, hematoma, infection, or dehiscence) at the three evaluation time points (hospital discharge, return to the hospital for removal of stitches, and the final postpartum follow-up visit) (Table 5).

Of the women who were found to have a C-section scar complication at the time of stitch removal (days 7–10), 14.1% and 10.6% of patients in the intervention and nonintervention groups, respectively, received antibiotics (cephalexin). Only one woman in the electrocautery group required hospitalization to drain a hematoma and resuture the wall. Another woman with a hematoma required no intervention.

## 4. Comment

Among women who had undergone zero to one previous C-section, no significant differences were found in surgical wound complications (seroma, hematoma, signs of infection, and dehiscence) at the three different time points (postpartum days 3-4, 7–10, and 30–40) between women submitted to C-section in whom electrocautery was used for homeostasis or coagulation and those in whom no electrocautery was used. Preparation of the medical team involved in this study included classes with printed material on care during hemostasis and the prevention of surgical infection, which may have had a positive effect on the results found in the two groups.

Unfortunately, we found no previous studies on the additional risk of surgical wound complications following C-section in which electrocautery for homeostasis was used for comparison of our results. The intervention evaluated in this study reduced the total time of the procedure by only five minutes, which was surprising. The C-section was expected to take much longer without the use of cautery because of the need for suture hemostasis. However, the total time of surgery was high in both groups, which can be explained by the fact that most of the C-sections were performed by year 2 and year 1 obstetrics and gynecology residents, who were expected to increase the duration of surgery by more than 50% [14].

Contrary to our expectations, all intraoperative clinical complications occurred in women in whom electrocautery was not used. On the other hand, the two hematomas found between days 7 and 10 occurred, surprisingly, in women in whom electrocautery had been used. The assumption that avoidance of cautery could be associated with a higher risk of hematoma certainly contributed towards the extreme care used in hemostatic suturing, thereby resulting in a reduction in the occurrence of hematomas in this group.

No statistically significant differences were found in the Apgar scores of the newborn infants in the two groups, and no complications occurred. These results were expected because the amount of electrical energy used in the mother was less than that used in fetal surgery procedures and lasted for a shorter period of time [9, 10]. Moreover, electrical energy must be disseminated to act directly on tissues [3], and the various layers of tissue and the placenta separating the fetus from the electrocauterizer may act as protection. Finally, biosafety regulations are implemented to guarantee that energy is grounded through the fastest route, thus not affecting the fetus [1].

The increase in postdischarge complications was similar in both groups and was in agreement with the postdischarge surveillance rates reported in other publications both in Brazil and abroad. Dantas [12] reported that 75% of signs of C-section scar infections become apparent between days 7 and 11, reaching 95% by day 14 after discharge. Creedly and Noy [15] reported an infection rate of 2.8% during hospitalization that increased to 17.0% when the women responded to a questionnaire 30 days after discharge. The infection rates in the electrocautery group (10.0%) and that in the nonintervention group (8.7%) at days 7 to 10 in the present randomized pilot study were lower than those reported by other national authors (14.4% and 17%, resp.), but were similar to those reported in other international publications [16–18].

Seromas were the most common complication in both groups; however, they occurred more frequently in the electrocautery group. This finding was expected because electrocautery promotes coagulation by breaking down molecules via lipolysis. Nevertheless, even without the use of this device, the incidence of seromas was high. Other factors, such as the thickness of subcutaneous tissue, may also be associated with this complication. The mean body mass index was similar in both groups and was indicative of a borderline overweight status, a fact that may have contributed to these rates.

Even with complications occurring in around one-fifth of C-section scars, all the women had recovered by day 40 postpartum and their scars had healed with no hematomas, seromas, or signs of infection. However, this pilot study evaluated only functional and not aesthetic aspects and failed to assess patient satisfaction with respect to the scar.

Findings may be different in other populations. In addition, other factors that could be associated with the presence of complications, such as maternal anaemia, were not evaluated. Nevertheless, this was a population of pregnant women with reasonable nutritional and educational status, with a mean body mass index compatible with overweight status, and in whom no greater risk of surgical scar complications was found with the use of electrocautery.

The results of this randomized study are based on the CONSORT guidelines [2, 19]. The principal limitation of this clinical trial was the lack of previous studies that would have permitted calculation of a sample size sufficient to confirm or discard the possibility of risks associated with the use of electrocautery in C-sections. Sample size had to be calculated based on a study of infectious surgical scar complications detected during postdischarge surveillance with no mention of cautery. The rates reported in this study were used to represent the nonintervention group, and it was assumed that the intervention would double the risk of complications.

Nevertheless, the incidence of infectious complications in the 224 women in the 2 groups was lower than expected, increasing the type I error. A 50% increase in the risk of complications was found, but it was not significant. Moreover, the rates of infection in the intervention and nonintervention groups were only 10.0% and 8.7%, respectively, which were lower than predicted based on previous studies on infection rates. Therefore, this randomized trial is a pilot study that may serve as a basis for the calculation of sample sizes in further studies in which greater statistical power may supply more conclusive results. Whether the use of electrocautery increases the incidence of complications in C-section scars remains unconfirmed despite a relative risk of 1.5, wide confidence interval, and difference is not considered to be statistically significant.

Because of these limitations, a definitive change in C-section guidelines regarding hemostasis cannot be recommended. Further studies with greater statistical power must be performed to increase the current knowledge on the use of electrocautery for both coagulation and tissue cutting in procedures performed in pregnant women. It is reasonable to suggest, however, that as recommended, the obstetrician should use electrocautery sparingly for coagulation during surgery, and only when suture hemostasis is contraindicated.

## Figures and Tables

**Table 1:** Demographic characteristics, parity, and prenatal care in pregnant women submitted to C-sections with and without the use of electrocautery.

Variable	With electrocautery				Without electrocautery			
	*n*	Mean	SD	Median	*n*	Mean	SD	Median
Age (years)	109	24.9	6.5	23.0	111	24.5	6.4	23.0
Schooling (years)	109	10.0	1.7	11.0	111	9.7	1.8	11.0
Pregnancies	109	1.79	1.00	2.00	111	2.01	1.17	2.00
Caesarean sections	109	0.34	0.48	0.00	111	0.38	0.49	0.00
Weight (kg) at first antenatal visit	109	64.9	16.2	61.0	111	66.2	15.6	64.0
Weight (kg) at final antenatal visit	109	76.8	16.9	73.0	111	77.3	15.3	76.0
Initial BMI	109	25.3	5.9	23.6	108	25.4	5.0	25.0
Gestational age (weeks)	108	39.1	1.8	39.4	107	39.0	1.7	39.3

BMI: body mass index; SD: standard deviation.

**Table 2:** Surgical scar complications on day 3 following C-section with and without the use of electrocautery.

	Treatment (use of electrocautery)			
	Yes		No	
	*N*	%	*N*	%
Hematoma				
No	109	100.0	111	100.0
Seroma				
No	108	99.1	111	100.0
Yes	1	0.9	0	
Infection				
No	106	97.2	111	100.0
Yes	3	2.8	3	

**Table 3:** Surgical scar complications between days 7 and 10 after C-section with and without the use of electrocautery.

	Treatment (use of electrocautery)			
	Yes		No	
	*n*	%	*n*	%
Hematoma				
No	98	98.0	104	100.0
Yes	2	2.0	0	
Seroma				
No	83	83.0	92	83.0
Yes	17	17.0	12	11.5
Infection				
No	90	90.0	95	91.3
Yes	10	10.0	9	8.7

**Table 4:** Surgical scar complications between days 30 and 40 following C-section with and without the use of electrocautery.

	Treatment (use of electrocautery)			
	Yes		No	
	*n*	%	*n*	%
Hematoma				
No	97	100.0	95	99.0
Yes			1	1.0
Seroma				
No	97	100.0	93	96.9
Yes			3	3.1
Infection				
No	97	100.0	93	96.9
Yes			3	3.1

**Table 5:** Accumulated surgical scar complications∗ assessed during follow-up, according to the use of electrocautery for hemostasis.

	Treatment (use of electrocautery)				*P*-value	RR
	Yes		No	
	*n*	%	*n*	%
Any complication prior to discharge						
No	106	97.2	111	100	0.1199	Not calculable
Yes	3	2.8	0			
Any complication up to postpartum days 7–10						
No	77	77.0	88	84.6	0.1667∗	1.50 (0.84–2.60)
Yes	23	23.0	16	15.4		
Any complication up to postpartum days 30–40						
No	77	77.0	78	81.3	0.5006∗	1.21 (0.69–2.11)
Yes	23	23.0	18	18.8		

*includes hematoma, seroma, dehiscence, and signs of infection.
